# Clinical, genetic, and pathological features of male pseudohermaphroditism in dog

**DOI:** 10.1186/1477-7827-9-12

**Published:** 2011-01-21

**Authors:** Enrico Bigliardi, Pietro Parma, Paolo Peressotti, Lisa De Lorenzi, Peter Wohlsein, Benedetta Passeri, Stefano Jottini, Anna Maria Cantoni

**Affiliations:** 1 Faculty of Veterinary Medicine, Department of Animal Health, Parma, Italy; 2 Private Clinics, Parma, Italy; 3 Faculty of Agronomy, Department of Animal Science, Milan, Italy; 4 University of Veterinary Medicine Hannover, Department of Pathology, Germany

## Abstract

Male pseudohermaphroditism is a sex differentiation disorder in which the gonads are testes and the genital ducts are incompletely masculinized. An 8 years old dog with normal male karyotype was referred for examination of external genitalia abnormalities. Adjacent to the vulva subcutaneous undescended testes were observed. The histology of the gonads revealed a Leydig and Sertoli cell neoplasia. The contemporaneous presence of testicular tissue, vulva, male karyotype were compatible with a male pseudohermaphrodite (MPH) condition.

## Background

Mammalian sexual development depends on the successful completion of a series of steps under genetic and hormonal control. This process requires the accomplishment of three steps: chromosomal sex, gonadal sex and phenotypic sex. At fertilization the chromosomal sex is determined and successively *XY *embryos will develop testes whereas *XX *ones will develop ovaries (gonadal sex). The process regulating gonadal development is under genetic control and is referred as sex determination. Once gonads are well developed and fully functional, hormones play a key role in phenotypic sex establishment. In male, embryonic testes produce two key hormones: anti-Muellerian hormone (AMH; also called mullerian-inhibiting substance [MIS] or factor [MIF]) and testosterone. These hormones, together with testosterone derivative dehydrotestosterone, are responsible for the development of internal and external male phenotype. In the *XX *embryos the absence of these hormones lead to female phenotypic sex development [1].

In mammals, the *SRY *gene represents the key gene for testis differentiation in the embryos. There is substantial evidence that this gene product is necessary and sufficient to induce the undifferentiated gonads to develop as testes [2]. Moreover, since its discovery the analyses of sex-reversal subjects leads to the identification of several other genes involved in sex determination process, and among them *Sox9*, *Wnt4 *and *Rspo1 *seems to play a major role [1].

Abnormal sex development could derive from sex determination errors (i.e. discordance between chromosomal and gonadic sex) or from discordance from gonadal and phenotypic sex (i.e. errors in sex differentiation process). In the first case the affected animals are referred as sex reversal, whereas in the second one they are called pseudohermaphrodites.

In small animals there are four principal categories of intersex individuals:

1) True hermaphroditism: in these animals there are both gonadal tissues, but the secondary sex characteristics and external genitalia of the opposite sex. Testes and ovaries are present in various combination. A testis may be found on one side and an ovary on the other side, an ovotestis or bilateral ovotestes may be present [3].

2) Female pseudohermaphroditism: these animals have *XX *chromosomes (without sign of *SRY *gene presence) and ovaries but external genitals appear masculine. The bitch has an enlarged clitoris and even a prostate. The cause is the excess of testosterone during pregnancy even if the real origin of this abnormal testosterone content is often unknown. Normally these subjects are sterile and ovariohysterectomy is recommended [4].

3) Male pseudohermaphroditism: the chromosomal gender is *XY *with an intact and functional *SRY *gene. The testes are present but the external genitals appear feminine. In several cases the dog has vestigial oviducts and uterus. The testes may be located within the abdomen, the scrotum or lateral to the vulva. A penis can be present or, more often, it is an enlarged clitoris. If penis and testes are present the diagnosis is more difficult and the abdominal surgery is necessary to find vestigial female organs [4].

4) Unclassified subjects: the animals that are included in this category do not fit into the other principal categories. For exact sex assignment it is necessary to test the chromosomes constitution and at last to verify *SRY *gene presence [5].

In veterinary medicine no cases of pseudohermaphroditism in association with testicular tumors are described. The aim of this paper is to describe the genetic, clinical and histopathological features of a male pseudohermaphrodite dog with interstitial (Leydig) cell testicular tumor.

## Methods

A mixed-breed 8-years old dog weighing 10 kg with intersex abnormalities was presented to the Veterinary Hospital of Parma University. A physical examination was carried out, blood samples were collected from the cephalic vein for cytogenetic and sex hormones analysis. Ultrasound examination was performed with a 7.5 Mhz probe (MyLab, Esaote Firenze, Italy). For the surgical removal of the testes, the dog was premedicated with atropine sulphate (Atropina solfato, Ati, Ozzano Emila Bologna Italy) 0,05 mg/kg, and the anaesthesia was induced using a mixture of ketamine (Imalgene100, Merial, Italia) 5 m/Kg and medetomidine (Domitor, Pfizer, Roma, Italy) 40 mcg/kg i.m.. The anaesthesia was maintained with isoflurane (2%), and oxygen was supplied by a cuffed endotracheal tube. Cephalexin (Mylan, Milano Italy) 15 mg/kg was administered at the time of induction. In the inguinal region two incisions laterally to the vulva were performed to remove the testes using a standard open orchiectomy technique. The postoperative treatment consisted of amoxicillin and clavulanic acid (Synulox Pfizer, Roma, Italy) 10 mg/kg and 2.5 mg/kg, respectively, every 12 hours for 7 days.

After surgical excision testicles were macroscopically examined, and fixed in 10% neutral buffered formalin for at least 3 days. For histological examination, several longitudinal slices of the testicles and several transversal sections of the spermatic funicles were processed histotechnologically according to standard laboratory procedures, cut at 5 μm, and stained with haematoxylin and eosin.

### Immunohistology

Immunohistology was performed with various murine monoclonal antibodies (all from Dako, Hamburg, Germany, unless otherwise stated). Cytokeratin (CK, clone AE1/AE3 against CK1 - 8, 10, 13 - 16, 19, diluted 1 in 500 in phosphate-buffered saline [PBS, pH 7.1]), vimentin (clone V9, diluted 1 in 100 in PBS), Melan A (clone A 103; diluted 1 in 600 in PBS), and p53 (clone DO-7; 1 in 50 in PBS). In addition, rabbit polyclonal antibody specific for c-kit (CD 117; 1 in 100 in PBS), diluted 1 in 200 in PBS was used. Briefly, after dewaxing, tissue sections were immersed in H_2_O_2 _0.5% in methanol for 20 min. Antigen retrieval was achieved by incubation in acid citric monohydrate solution (pH 6.2; 0.01 M) for 25 minutes in a microwave oven (800 W). Non-specific binding was blocked with inactivated goat serum, diluted 1 in 5 in PBS. This was then replaced by the primary antibody before incubation in a moist chamber at 4°C overnight. After washing, tissue sections were incubated with a biotin-labelled goat-anti mouse or rabbit IgG diluted 1 in 200 in PBS (Vector Laboratories, Burlingame, CA, USA). The avidin-biotin-peroxidase method (Vector Laboratories) was then applied according to the manufacturer's instructions. The chromogen used was 3, 3'-diaminobenzidine-tetrahydrochloride (Sigma Chemie, Taufkirchen, Germany) 0.05% with H_2_O_2 _0.03% as substrate in 0.1 M Tris-buffered saline (Tris-hydroxymethyl-aminomethane; Merck, Darmstadt, Germany), pH 7.6. Tissue sections were counterstained with Mayer's haematoxylin and mounted. Skin, a malignant melanoma, a squamous cell carcinoma and a mastocytoma from a dog were used as positive controls. For negative control purposes, the primary antibody was replaced by ascitic fluid from non-immunized Balb/cJ mice (Biologo, Kronshagen, Germany) or rabbit serum (Sigma Chemie, Taufkirchen, Germany).

### Cytogenetics and *SRY *amplification sequencing

GIEMSA stained metaphases, obtained from peripheral blood lymphocyte cultures, were obtained following standard methods [6], whereas dog *SRY *gene was amplified and sequenced as reported by De Lorenzi and colleagues [7]. Briefly whole Dog *SRY *coding region (GenBank AF107021) was amplified using the following primers (5'-3'): *SRY*-Dog-F: ctttccaacttccctccgta, *SRY*-Dog-R: ggacgtttcgttagccagag. The PCR product was 813 bp long. PCR was performed using AmpliTaq Gold DNA Polymerase (Applied Biosystems). PCR product was purified and sequenced as described by Parma and colleagues [8].

## Results

**Figure 1:**
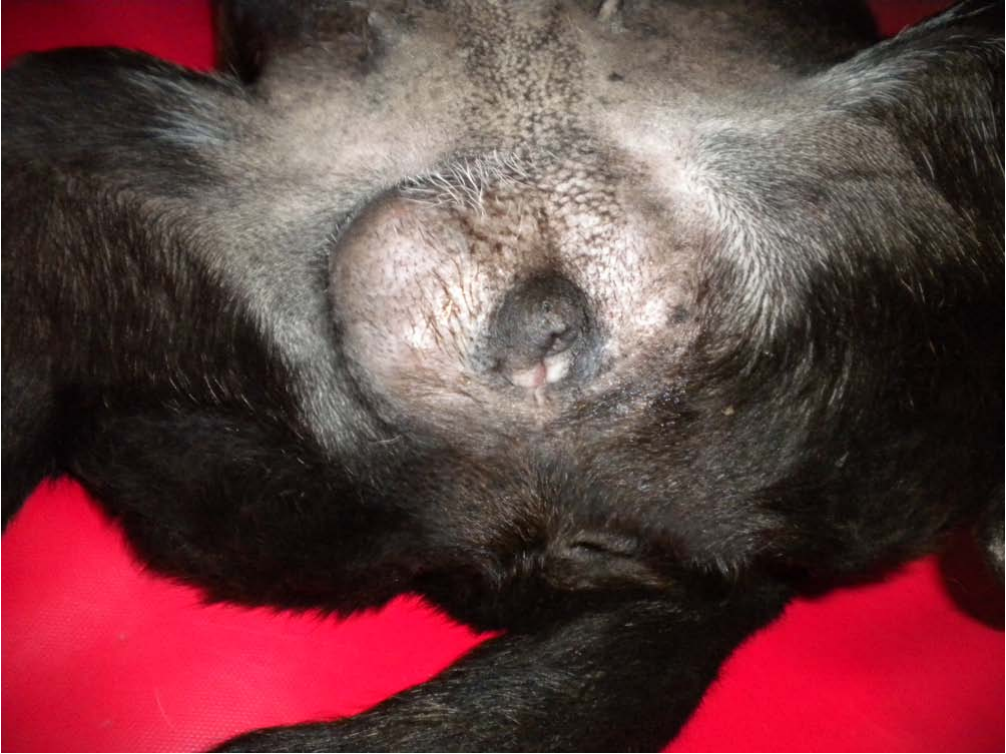
**External genitalia: the vulva is present and none structure compatible with the penis and scrotum are visible**. Lateral to the vulva, on the right and left sides, two enlarged, variable size, subcutaneous structures are identifiable (neoplastic testicles).

Physical examination of the external genitalia revealed the presence of a vulva, the absence of a scrotum and penis. Laterally to the vulva, on the right side, an enlarged subcutaneous structure with irregular surface was found. On the left side a small and regular subcutaneous structure (Figure 1) was identified. The ultrasound examination of these structures were compatible with right and left testis. No evidence of scrotum was found. Abdominal ultrasound did not show uterus or other Mullerian derivatives. Laparotomy for ovariohysterectomy was performed seven years earlier, but neither a uterus nor the ovaries were found. Most likely, the presence of undescended testes was considered.

### Ultrasonography

**Figure 2:**
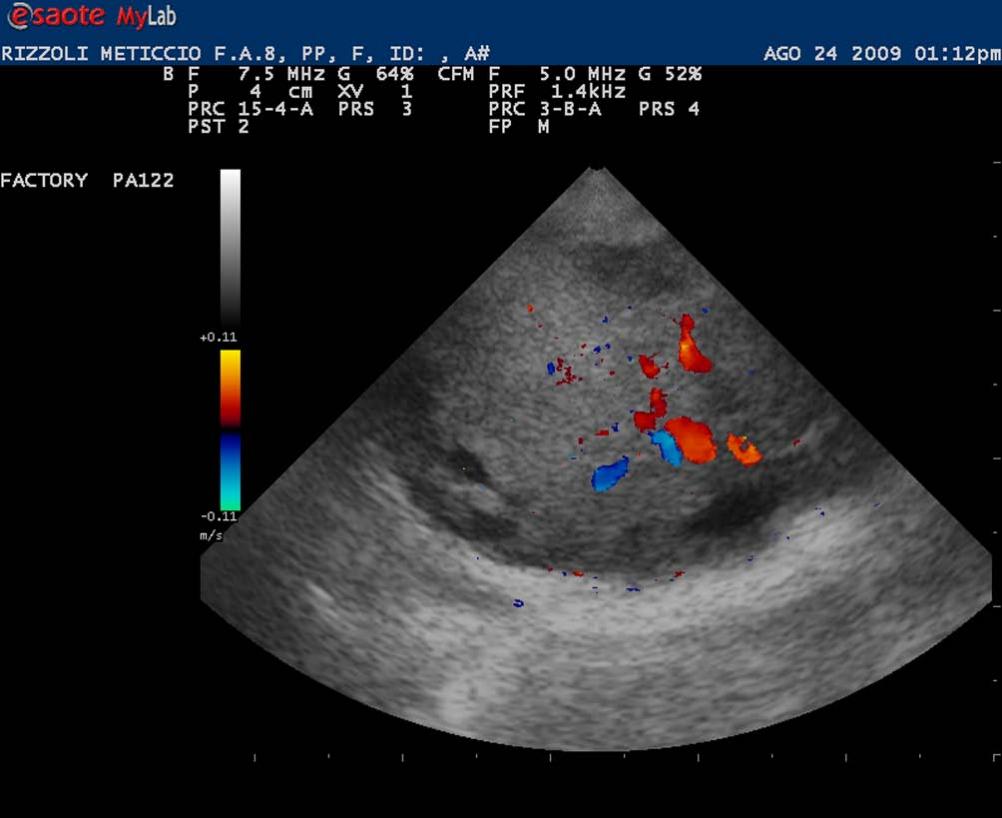
**Ultrasonographic and colour doppler images of right testis: the normal echographic pattern of testis is not recognizable**. The mediastinum testis is not showed and blood flows is visible within the parenchyma.

**Figure 3:**
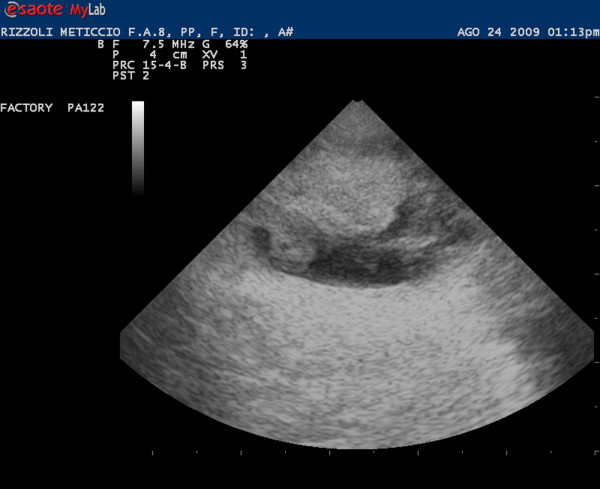
**Ultrasonographic image of left testis: The testis is small and iperechoic**. None Doppler signal are present and it appears as fat tissue.

The ultrasound examination of right testis showed a complex mass with a necrotic area. The colour Doppler showed an increase of peripheral and inner signal of blood flows (Figure 2). The left testis was not well defined and it appeared hyperechoic as fat tissue without evidence of blood flow (Figure 3).

### Cytogenetic analysis and *SRY *gene analysis

**Figure 4:**
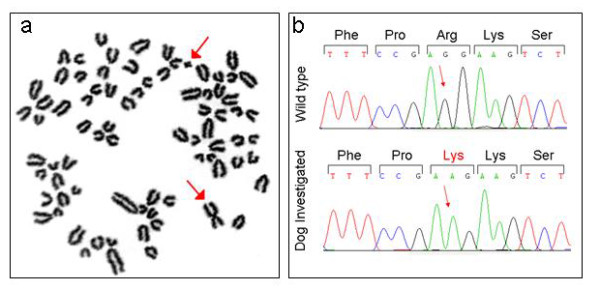
**Metaphase of the analysed subject**. Sex chromosomes are indicate by the red arrows.

Cytogenetic investigation revealed a normal male karyotype (2n = 78, *XY*) on all observed metaphase plates (more than 50, Figure 4). The analyses of *SRY *gene revealed the presence of a bp change (bp 68 of dog *SRY *coding sequence corresponding to bp323 of AF107021 GenBank sequence G > A). This change leads to a Arg > Lys change (amino acids (AA) 23 of protein sequence).

### Pathomorphological and immunohistological findings

**Figure 5:**
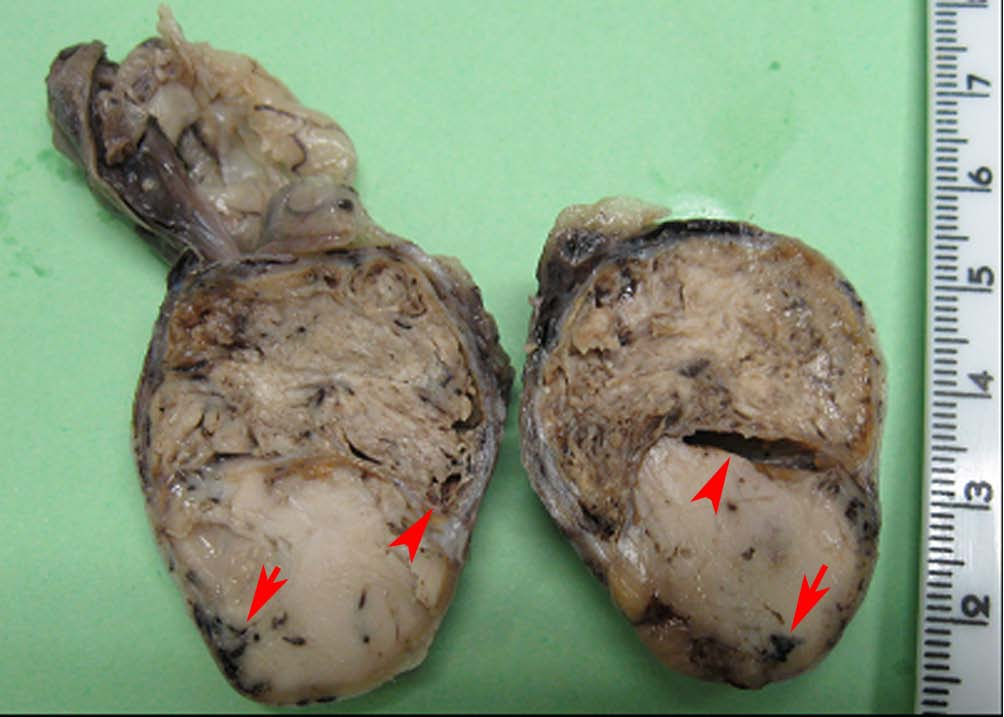
**Right testicle, interstitial [Leydig] cell tumor**. Neoplastic proliferation with wrinkled (top) and lardaceous (bottom) aspect, yellowish colour, multifocal haemorrhages (arrows) and cystic formations (arrowheads), replaces the whole testicular parenchyma. At the top is also visible the spermatic funicle.

Macroscopically, the right testicle was coiled by a thin, translucent tunica resembling the albuginea. Beneath the tunica prominent superficial veins were visible. The testicle appeared spherical with corrugated surface, firm and whitish. At the cranial pole, vessels and the spermatic funicle surrounded by connective tissue, mesothelium and scarce adipose tissue, appeared like a cord structure extending to the medial part of the epididymal testicular margin. One centimeter down from the cranial pole, on the free surface of the testicle, another cord-like structure of vessels, ducts and striated muscle surrounded by connective tissue originated from the testicular capsule. On cut section, there was a thin tunica albuginea which surrounded completely the parenchyma separated in two identical parts by filamentous connective tissue. After fixation, the testicular parenchyma near the cranial pole was uniformly whitish-yellowish, lardaceous, with small circular reddish areas (multifocal haemorrhages). The testicular parenchyma at the cranial pole had yellowish colour, multifocal haemorrhages, a wrinkled aspect, was less consistent and had small cystic formations (Figure 5).

**Figure 6:**
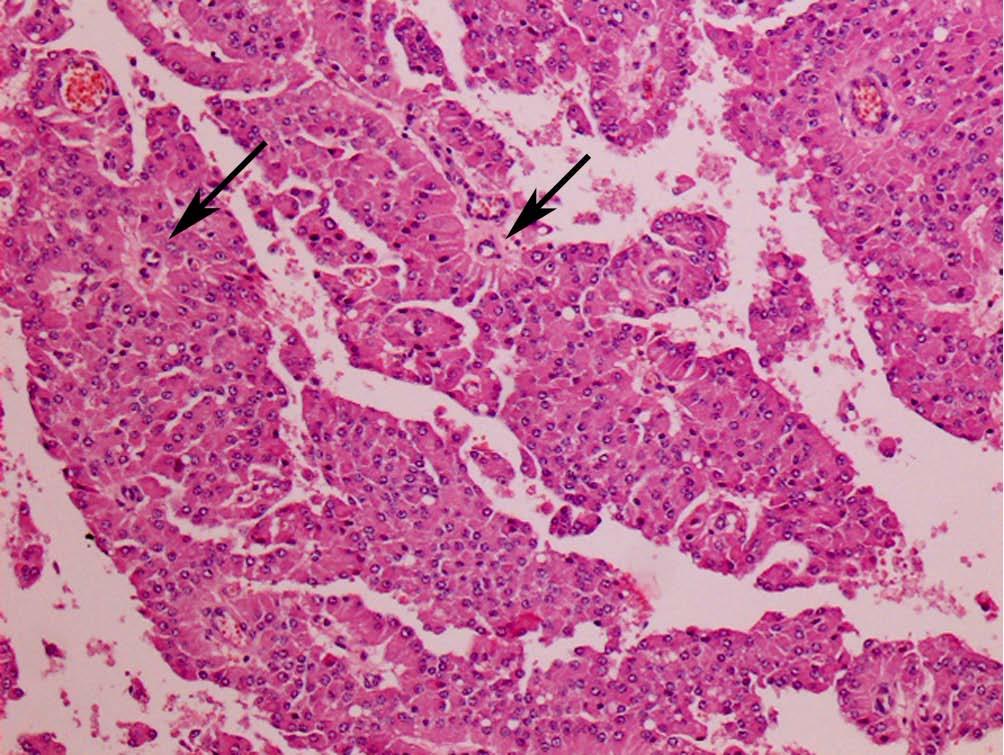
**Right testicle, interstitial [Leydig] cell tumor, numerous cells with polyhedric shape and eosinophilic cytoplasm, arranged radially around blood vessels (pseudorosettes) (arrows) are visible**. Neoplastic cells are supported by a thin connective stroma Haematoxylin and Eosin, 10X.

Histologically, a neoplasia of 1.5 × 2.5 × 2.0 cm, densely cellulated and well circumscribed by the testicular albuginea and the visceral lamina was observed (Figure 6). Proliferating cells were arranged in cords and occasionally radially disposed around the blood vessels in pseudorosette formations supported by a thin connective stroma. Tumour cells had a polyhedric shape, distinct cell borders, abundant eosinophilic or microvacuolated cytoplasm with a moderately increased nucleus/cytoplasm (N/C) ratio. Nuclei were round, centrally located, with granular chromatin and a single round nucleolus. Anisocytosis, anisokaryosis and karyomegaly were marked. One or two mitotic figures were observed per high power field. Neoplastic cell cords were occasionally separated by cysts containing homogeneous and lightly eosinophilic material and small haemorrhagic areas. Subcapsularly, some atrophic and compressed seminiferous tubules without spermatogenesis were found.

The two cord structures were composed of rare tubular structures lined by columnar pseudostratified epithelium with mostly columnar cells, surrounded by soft connective tissue and smooth muscle cells (epididymis). Fibrous connective tissue, numerous vessels and adipose tissue surrounded the epididymal tubules. These structure were consistent with mesothelium (visceral lamina) and striated muscle fibers (cremaster muscle). In addition, rare vascular structures surrounded by connective tissue consistent with the pampiniform plexus, adipose tissue and mesothelium (visceral lamina) were observed. There was no evidence of the deferens duct.

**Figure 7:**
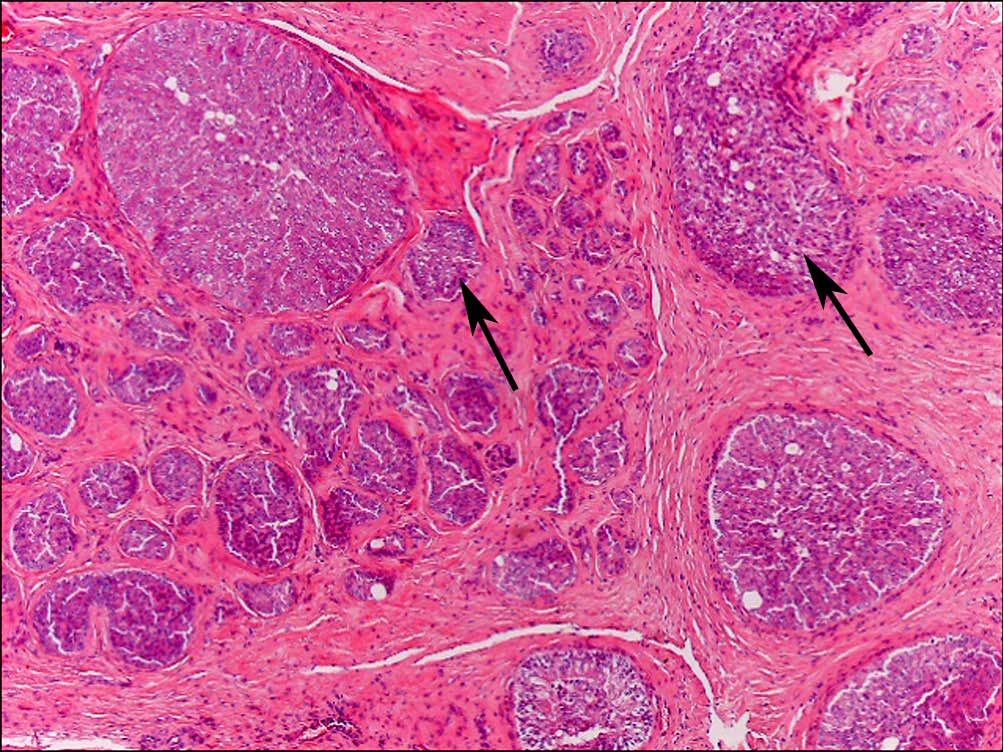
**Left testicle, sustentacular [Sertoli] cell tumor**. Hypoplastic and neoplastic seminiferous tubuli surrounded by dense connective tissue are visible. The tubules are dilated and lined by columnar cells, rarely microvacuolated, and by numerous large cells with poorly distinct borders, eosinophilic and macrovacuolated cytoplasm (sustentacular neoplastic cells, arrows). Haematoxylin and Eosin, 4X.

The left testicle was atrophic and covered by a thin, translucent tunica resembling macroscopically the tunica albuginea. The testicle was spherical, firm, with a smooth and grayish surface. On cut section at the cranial pole there was a gray-yellowish, lardaceous structure with a thin cord of about 1 mm in diameter inside, probably an atrophic spermatic cord. Histologically, the lobules were immersed in a dense connective tissue with hypoplastic seminiferous tubules. The tubules were lined by columnar cells, rarely microvacuolated, resembling Sertoli cells (Figure 7). The lumina were dilated by numerous large cells with poorly distinct borders, eosinophilic or macrovacuolated cytoplasm, voluminous round nucleus with vesicular chromatin and one nucleolus. There was moderate anisocytosis, anisokaryosis and karyomegaly. One or two mitotic figures were found per high power field.

On transversal sections of the lardaceous area near the cranial pole of the testicle, outside of the testicular tissue, there were rare tubular structures lined by cuboidal cells forming a pseudostratified epithelium surrounded by soft connective tissue and smooth muscle cells (epididymis), and abundant adipose tissue, rare vascular structures and rare striated muscle cells (cremaster muscle). There was no evidence of the deferens duct.

Immunohistochemistry performed on the right testicle revealed a focally faint immunostaining of CK in small groups of neoplastic cells. There was a multifocal slight expression of vimentin in neoplastic cells. No immunostaining was observed using the antibodies against Melan A, c-kit and p53. The neoplasia of the left testicle revealed a moderate vimentin expression. In addition, there was a focally marked immunostaining of CK in neoplastic cells. Few cells located at the periphery of seminipherous tubules interpreted as Sertoli cells expressed Melan A. Immunostaining was not observed using antibodies specific for c-kit and p 53 within the neoplasia.

According to the morphological findings the tumours were classified as an interstitial [Leydig] cell tumor with solid-diffuse and cystic-vascular patterns (right testicle) and a Sertoli [sustentacular] cell tumor (left testicle) [9].

## Discussion

Male pseudohermaphroditism is a condition of sex differentiation disorder in which the gonads are testes and the genital ducts or external genitalia are incompletely masculinised [10]. In the present case the dog was classified as male pseudohermaphrodite, because testes, vulva and the *SRY *gene, together with a 78, *XY *chromosome complement, were present, even if in this gene a new SNP (Single Nucleotide Polymorphism) was identified. The *SNP *discovered is not reported in any public database or in any publication involving dog *SRY *gene studies (Figure 4). To establish if a *SNP*, leading to AA change, is responsible or not of a abnormal phenotype it is no an easy task. About this *SNP *we can observe that: a) the change occurs outside the *HMG *box and it is clear that this domain represents the unique important functional domain of this gene (almost all *SRY *missense mutations leading to abnormal sex development are present in this region and those located upstream to this functional domain, as reported here, are frameshift mutation); [11]; b) The Arg to Lys AA substitution is not a dramatic chance (both are basic and hydrophilic residues), even if very few cases of the same kind of mutation leading to abnormal phenotypes are reported for other genes [12,13]; c) Bioinformatic analysis, using PolyPhen software [14], of this mutation suggests a benign effect [15-17]. One possible solution can be the *SRY *sequence analysis on a large number of normal male dog in order to establish the real nature of this *SNP *(rare polymorphism or mutation?) but in this case we can consider that it is possible a not fully expression of the mutation (as this condition is yet reported in human cases of *SRY *missense mutation) so we can observe the *SNP *in normal dog. Nevertheless our hypothesis is that this *SRY SNP *could not be responsible for the observed phenotype.

In addition to the pseudohermaphroditism in the described case, neoplastic growth was found in both testicles, an interstitial cells tumour and a Sertoli cells tumor. Already at ultrasonograpic examination, the increase of blood flow was suggestive of a neoplastic growth [18]. Only few cases of testicular tumors (dysgerminomas and Sertoli-Leydig cell tumor) in phenotypic female (*XX*) patients are described in human medicine [19], probably because they are early recognized and treated. In the veterinary literature, one case of interstitial cell hyperplasia and one case of Sertoli cell neoplasia in dogs with Müllerian duct syndrome and one case of an interstitial cell tumour in an intersex goat are described [20-22]. To the best of the authors knowledge, this is the first report of an interstitial and Sertoli cell in a male pseudohermaphrodite dog.

Retained testes lack spermatogenesis and have an increased risk of tumor development [23], particularly the seminoma and the Sertoli cell tumor [24]. In this case the testicles were retained subcutaneously in the perineal region for the whole life of the animal and this could have contributed to the development of the Sertoli cell neoplasia but not of the Leydig cell tumor, because cryptorchidism seems not to be a predisposing factor for interstitial cell neoplasm [24].

Due to the good differentiation of both testicular tumours, immunohistochemistry was only performed for vimentin, CK and Melan A. Histological appearance of most testicular tumours in dogs is highly specific and immunophenotyping of the neoplasia is not necessary. In cases of mixed tumours or poorly differentiated testicular neoplasias the use of 3-beta-hydroxysteroid dehydrogenase (3beta-HSD) and LH receptor markers may help in the identification of the tumour type [25]. Sertoli and Leydig cell tumors are usually benign and rarely metastasize, however both can lead to the development of hyperestrogenism related problems (feminization of male dogs) such as: gynaecomastia, penis atrophy, bone marrow suppression, alopecia, thinning and hyperpigmentation of the skin [24]. In this case lesions of hyperestrogenism were not present.

Descent of testes, epididymis and spermatic cord is a complex series of events that requires hormonal, constitutive, and nervous control. This process occurs in three main stages: the relative transabdominal migration phase, the intra-inguinal phase and extra-inguinal migration [23]. In this case the process stopped in the 3^rd ^phase. This may have occurred due to the lack of the scrotum, the particular conformation of the whole genital system and the hormonal metabolism of the dog.

Examination of the testes revealed the presence of inactive seminiferous tubules and epididymes. In this case there were atrophic seminiferous tubules and epididymes, which is probably due to the compression of the tumour on the surrounding testicular parenchyma.

Both testicles also presented a complete tunica albuginea, epididymes, vessels and cremaster muscle but no deferent ducts, probably because the surgical exeresis did not include it.

## Conclusion

This results confirm that when a congenital problem is suspected, karyotyping should be performed before any other test and if the reproductive problem of intersex is confirmed, surgical resection of genital tract is the treatment of choice to avoid clinical problems and the development of neoplastic diseases [26].

## Competing interests

The authors declare that they have no competing interests.

## Authors' contributions

BE performed the clinical and ultrasonographic examination, conceived, designed and wrote the paper. PaP and DL carried out the genetic analysis and helped to draft the manuscript. PeP performed the clinical examination and surgery procedures. WP, JS and PB performed the histological and immunohistochemical examination, intellectually contributed and helped to write the paper. CAM was responsible for the histological procedures and reviewed the paper. All authors read and approved the final manuscript.
